# Diversity of Oral Microbiome of Women From Urban and Rural Areas of Indonesia: A Pilot Study

**DOI:** 10.3389/froh.2021.738306

**Published:** 2021-11-29

**Authors:** Armelia Sari Widyarman, Citra Fragrantia Theodorea, Nadeeka S. Udawatte, Aradhea Monica Drestia, Endang W. Bachtiar, Tri Erri Astoeti, Boy M. Bachtiar

**Affiliations:** ^1^Department Head of Microbiology, Faculty of Dentistry, Trisakti University, West Jakarta, Indonesia; ^2^Department of Oral Biology, Faculty of Dentistry, Universitas Indonesia, Central Jakarta, Indonesia; ^3^Singapore Oral Microbiomics Initiative, National Dental Research Institute Singapore (NDRIS) National Dental Centre Singapore, Oral Health ACP, Duke NUS Medical School, Singapore, Singapore; ^4^MiCORE Laboratory, Faculty of Dentistry, Trisakti University, West Jakarta, Indonesia; ^5^Department Preventive and Public Health Dentistry, Faculty of Dentistry, Trisakti University, West Jakarta, Indonesia

**Keywords:** oral microbiome, women, urban-rural areas, Indonesia, pilot study

## Abstract

**Objective:** The studies on the influence of geographical and socio-economic factors on the oral microbiome remain underrepresented. The Indonesia basic health research (RISKESDAS) 2018, showed an increasing trend in non-communicable diseases compared with the previous report in 2013. The prevalence of diabetes, heart disease, hypertension, and obesity are reported to be higher in urban areas than in rural areas. Interestingly, non-communicable diseases were found to be more prevalent in women than men. This pilot study aimed to examine the oral health and oral microbiome derived from tongue samples of healthy Indonesian women from urban and rural areas.

**Methods:** Twenty women aged 21–47 years old from West Jakarta, residents of DKI Jakarta (*n* = 10) as representative of the urban area, and residents of Ende, Nangapanda, East Nusa Tenggara (*n* = 10) as representative of the rural area were recruited for this pilot study. The participants were evaluated by the Simplified Oral Hygiene Index (OHI-S) according to the criteria of Greene and Vermillion and divided into three groups. High-throughput DNA sequencing was performed on an Illumina iSeq 100 platform.

**Results:** The principal component analysis displayed a marked difference in the bacterial community profiles between the urban and rural localities. The presence of manifest was associated with increased diversity and an altered oral bacterial community profile in the urban women. Two bacterial taxa were present at significantly higher levels (adjusted *p* < 0.01) in the urban oral microflora (Genus *Prevotella* and *Leptotricia*) could account for this difference irrespective of the individual oral hygiene status. The linear discriminant analysis effect size (LEfSe) analysis revealed several distinct urban biomarkers. At the species level, *Leptotrichia wadei, Prevotella melaninogenica, Prevotella jejuni*, and *P. histicola*, show an excellent discriminatory potential for distinguishing the oral microflora in women between urban and rural areas. Further, using SparCC co-occurrence network analysis, the co-occurrence pattern in the dominant core oral microbiome assembly was observed to be specific to its ecological niche between two populations.

**Conclusions:** This is the first pilot study demonstrating the characterization of the oral microbiome in Indonesian women in urban and rural areas. We found that the oral microbiome in women displays distinct patterns consistent with geographic locality. The specific characterization of the microbiota of Indonesian women is likely linked to geographical specific dietary habits, cultural habits, and socio-economic status or the population studied.

## Introduction

Traditionally, 700 species of bacteria were thought to inhabit the oral cavity [1]. However, based on high-throughput DNA sequencing, it has now been determined that 19,000 phylotypes inhabit the oral cavity, including uncultivatable bacteria [2]. Previous studies have reported that the oral microbiomes in health and disease, including periodontitis and dental caries [3–5], may be linked to certain non-communicable diseases, such as heart disease, stroke, cancer, chronic respiratory disease, and diabetes [6–10]. The relationship between the microbiome and systemic diseases can be attributed to several factors such as the genetic makeup and oral hygiene status of an individual [1], geographical, dietary, and socio-economical, as well as lifestyle factors [1].

Indonesia is the most populous country in Southeast Asia; it encompasses one of the four most populous nations in the world. In 2020, its population stood at 273,523,615 people from various ethnic groups [11]. In fact, in 2007, the number of ethnic groups that comprise the population of Indonesia was estimated to be 633 [12, 13]. These groups, which all have different cultures and lifestyles, are spread across the 34 provinces of the country [12, 13]. Oral diseases like periodontitis, caries, and even systemic diseases like diabetes mellitus [14], cardiovascular diseases [15], and rheumatoid arthritis [16] are known to be associated with an imbalance in the oral microbiome composition. In Indonesia, the prevalence of diabetes, heart diseases, and rheumatoid arthritis were higher in residents of urban areas (1.9, 1.6, and 0.3%, respectively) than in residents of rural areas (1, 1.3, and 0.2%, respectively). Interestingly, all the foregoing preventable non-communicable diseases were more prevalent in women than in men [13]. Among oral diseases with high prevalence in Indonesia are caries [17] and the prevalence of periodontitis has also been at the higher end (73.1%) [18]. Perhaps, the susceptibility to these diseases often shows ethnic biases with a prominent number of diversified populations within a country, which inspired us to explore the geographical variations in the oral microbiome and its potential impact on oral health. Previous studies have demonstrated the ethnicity-specific clustering of microbial communities in saliva and subgingival biofilms in genetically different populations of different countries [19, 20]. In fact, substantial differences had been observed in the oral microbiome diversity among African groups, which may have been attributed to the difference in ancient subsistence pattern, lifestyle, diet, and prevalence of dental caries [19, 21]. Thus, it is conspicuous that geography represents an ensemble of genetic, environmental, and cultural factors that had played a role to sculpt the human oral microbiome architecture.

Interestingly, some studies have reported gender-specific significance with periodontal health status in the urban population of Indonesia; with a lesser presence of calculus deposition and higher presence of deep pockets in women based on the Community Periodontal Index of Treatment Needs (CPITN) criteria [22]. On average, women engage in lower levels of physical activity than their male counterparts [23]. Moreover, in terms of the amount of time spent on household activities (i.e., mild to moderate physical activity), rural women have been found to engage in higher levels of physical activity than their urban counterparts [24, 25]. In addition, the caloric intake of women is also more likely to change (either increase or decrease) throughout their lifetime [26]. These lower levels of physical activity and altered caloric intake are directly linked to the incidence of non-communicable diseases [27]. It is also important to recognize that the characteristics of modern lifestyles, including easy access to calorie-dense food, lack of exercise, and excessive sedentary behavior can all contribute to the development of certain diseases [28].

Determining the inter-batch technical effects of inter-population biology based on comprehensive observations of gender-, geography-, ethnicity-, and lifestyle-specific variations in the compositions of healthy microbiomes is challenging [29]. However, the characterization of microbial profiles is vital concerning identifying and correcting the microbial configurations implicated in diseases as well as understanding the properties of healthy microbiomes in different microbial ecologies.

The variations in microbial compositions in different cultural backgrounds have previously been investigated in several countries [29]. However, to our knowledge, there are no studies performed to examine the effect of geographical, socio-economic factors on oral health and oral microbiome in Indonesia. Therefore, this pilot study aimed to examine the oral health and oral microbiomes isolated from the tongue samples of healthy urban- and rural-dwelling Indonesian women.

## Materials and Methods

### Ethical Statement

This study was approved by the Ethical Committee of the Faculty of Medicine, Universitas Indonesia (1060/UN2.F1/ETIK/PPM.00.02/2019). All the subjects voluntarily participated in the study. The subjects provided written informed consent concerning all the components of the study. Further, all the study procedures were conducted in accordance with the requirements of the Helsinki Declaration regarding research on human subjects.

### Subjects

A total of 20 women (age range: 20–45 years) participated in this pilot study. Women from Jakarta capital city (*n* = 10) were considered representative subjects from urban areas whilst women from Ende, Nangapanda, East Nusa Tenggara (*n* = 10) were considered representative subjects from rural areas. The inclusion criteria were female Indonesian citizens who have not used an antibiotic or antihistamine and have not received any periodontal treatment for the last 3 months. The exclusion criteria for the study were previous gastrointestinal surgery, use of antibiotics within the last 3 months, smoking, use of prebiotics/probiotics, vegetarianism or veganism, nutritional or ergogenic supplements, and pregnancy or lactation. The participants were evaluated using the Simplified Oral Hygiene Index (OHIS) according to the criteria suggested by Greene and Vermillion [30] and then divided into three groups: poor, moderate, good based on their oral health. Following the oral hygiene assessment, tongue samples were collected from the participants.

### Tongue Swab Collection

For the tongue sample collection, a sterile cotton swab was wiped across the dorsum region of the tongue of each participant. This process was repeated (duplication), and the cotton swabs were then each placed into a 1.5-ml centrifuge tube containing 1 ml of sterile phosphate buffer saline (PBS) solution.

### Sample Collection and DNA Preparation

The genomic DNA was extracted from each tongue sample using a QIAamp DNA Mini Kit (Qiagen, Hilden, Germany) according to the instructions of the manufacturer. The concentration and purity of each DNA sample were determined using an Invitrogen Qubit 3.0 Fluorometer (Invitrogen, Carlsbad, California, United States) as well as other methods, including PCR amplification, the purification of the PCR products, library preparation, and sequencing, which will be described further below. All DNA samples from each group were labeled, pooled, and then mixed together well in a single tube.

PCR amplifications were performed for library preparation before sequencing. Primers were specifically designed to target the V3–V4 region of 16S recombinant DNA (rDNA) and modified with Illumina overhang adapters was amplified by PCR using the primers F (5′ -TCGTCGGCAGCGTCAGATGTGTATAAGAGACAGCCTACGGGNGGC WGCAG-3′), R (5′ GTCTCGTGGGCTCGGAGATGTGTATAAGAGACAGG ACTACHVGGGTATCTAATCC−3′). The PCR amplification was performed in a total volume of 25 μl, which contained 2.5 μl of genomic DNA (5 ng/μl), 1 μM of forward and reverse primers (5 μl of each), and 12.5 μl of 2X KAPA HiFi HotStart ReadyMix (KAPA Biosystems, Boston, Massachusetts, United States). The PCR conditions were as follows: 95°C for 3 min as the initial denaturation step, followed by 25 cycles of 30 s at 95°C, 25 cycles of 30 s at 55°C, and 25 cycles of 30 s at 72°C. The amplicons were visualized through gel electrophoresis using 1% (w/v) agarose gel and 1X Tris-Acetate-Ethylenediaminetetraacetic acid (TAE) running buffer at 110 V for 15 min. The band was observed at 550 bp.

The first PCR clean-up step, which involved purifying the V3- and V4-region primer pairs of the 16S ribosomal RNA (rRNA) samples from the free primers and any other primer dimer species, was performed using AMPure XP Beads (Beckman Coulter, Brea, California, United States) in accordance with the Illumina iSeq 100 protocols for amplicon preparation. A PCR indexing was performed to connect the dual indices and Illumina sequencing adapters using an Integrated DNA Technologies (IDT) for Illumina Nextera UD Indexes Kit (Illumina, San Diego, California, United States). More specifically, the indexing was performed with 5 μl of DNA samples from the first clean-up PCR step, 10 μl of Nextera DNA UD Indexes Set A, 25 μl of 2X KAPA HiFi HotStart ReadyMix (KAPA Biosystems, Boston, Massachusetts, United States), and 10 μl of PCR-grade water (a total volume of 50 μl).

Using the AMPure XP Beads, the second PCR clean-up step was performed to clean the final library prior to quantification. The secondary visualization of the PCR products was achieved by means of gel electrophoresis (1% [w/v] agarose gel and 1X TAE running buffer at 110 V for 15 min). During this step, the band was observed at 630 bp. The pre-paration and sequencing of the final library were performed using the Illumina iSeq 100 platform at the MiCORE Laboratory, Faculty of Dentistry, Trisakti University, Jakarta.

### Sequence Curation and Annotation

Sequencing runs were first analyzed in Illumina Sequencing Analysis Viewer version 2.4.7 for analytical run Quality Control (QC) checks. If the QC metrics passed, the FASTQ files from the iSeq 100 were uploaded to the Galaxy web platform, using a public server (https://usegalaxy.org) [31]. Sample-specific QC metrics were identified using FASTQC version 0.72 (Galaxy, http://www.bioinformatics.babrahamac.uk/projects/fastqc). High-quality samples were then aligned to their respective reference sequence with bowtie2 [32] version 2.3.4.3 (Galaxy) using default parameters. Sequencing depth was determined using Genome Analysis Tool Kit depth of coverage on BAM files [33]. Local Run Manager v2.0, the 16S Metagenomics workflow performs taxonomic classification of 16S rRNA targeted amplicon reads using Illumina-curated version of the Greengenes database (greengenes.secondgenome.com/downloads/database/13_5), generated Total of 6,12,962 reads were clustered into operational taxonomic units (OTUs) at 3% divergence (97% similarity) and 1,317 OTUs were taxonomically classified. The resulting accumulation curves showed reasonable sequence saturation at a regional level (Supplementary Figure 1). The raw data has been registered for a public repository at BioProject ID PRJNA745286 (https://www.ncbi.nlm.nih.gov/bioproject/745286).

### Microbiota Data Analysis

The alpha diversity (Chao1, observed richness), together with rarefaction curves were calculated and visualized using the MicrobiomeAnalystR Platform [“phyloseq” and “ggplot” and “microbiomeseq” packages [34, 35] codes available at GitHub (https://github.com/xia-lab/MicrobiomeAnalystR)].

The beta diversity was also assessed with the respective algorithms implemented in the MicrobiomeAnalystR package and the evaluation of the community structure across the sample groups was assisted by non-metric multidimensional scaling (NMDS) plot calculated using the Bray–Curtis index of similarity retrieved from sample pairwise comparisons. A stress level of 0.20 was considered acceptable for the NMDS plots. Significance testing was performed using the permutational multivariate ANOVA (PERMANOVA) [36] test. The microbial community similarities and the homogeneity of dispersion between the rural and urban sample groups were tested using the Analysis of Similarity (ANOSIM) [37, 38].

SparCC algorithm [39], a network inference tool designed for compositional data, was used to estimate the microbial associations between the urban and rural populations and with their oral OHI status (OHIS). The SparCC algorithm uses a log-ratio transformation and performs multiple iterations to identify taxa pairs (genera) that are outliers to background correlations. The Sparse Inverse Covariance Estimation for Ecological Association and Statistical Inference (SPIEC-EASI) uses graphical network models [40] to infer the entire correlation network at once. Only correlations with an R-corr absolute value >0.6 and a *p* < 0.05 were plotted. We considered a valid co-occurrence event to be a robust correlation if Spearman's correlation coefficient was both >0.6 (or <-0.6) and statistically significant ([[I]]P[[/I]] <0.05). SparCC was used to generate true correlation coefficients from which pseudo-*p*-values were calculated using the MicrobiomeAnalystR Platform. The STAMP statistical software [41] and the Fisher's exact for multiple sample groupings were used with a cutoff of a corrected (Story false discovery rate (FDR) correction) *P* <0.01 to detect species with the most statistically significant differences between two localities. The results were filtered using a q-value of 0.05 and an effective size of 0.05 threshold in STAMP.

The potential biomarker taxa which differed in abundance and occurrence between the two geographic groups were detected by linear discriminant analysis (LDA) effect size (LEfSe) [42]. The LEfSe was calculated using the online Galaxy web application [43] with the Huttenhower lab's tool (https://galaxyproject.org/learn/visualization/custom/lefse/). First, the non-parametric factorial Kruskal–Wallis sum rank tests (alpha = 0.01) were used to detect the differential abundant features (genera) within the two geographic locations (rural and urban). Then, the effect size of each differentially abundant feature was estimated using the LDA. The all-against-one classes were compared (most stringent) and a linear discriminant analysis score value of 2.0 was chosen as the threshold for discriminative features. The DESeq2 package [44] in MicrobiomeAnalystR was used to identify the bacteria with the most significant changes in differential abundance at the phylum, genera, and species level. The relative abundance >0.1% and presence in >50% of the samples in at least one group were used for the above analyses unless otherwise specified.

### Statistical Analysis

Two-tailed Mann–Whitney tests and the Kruskal-Wallis tests were used to compare the continuous and categorical variables, respectively. The differential abundances were measured with the log2 fold change value, and multiple comparisons were corrected using the Benjamini–Hochberg correction (Q parameter = 0.1, FDR < 10%) [45]. The differential-abundance measurements were statistically significant if the adjusted *p*-value was < 0.05.

## Results

### Study Group Characteristics and Oral Microbiome Dysbiosis Between Urban and Rural Locations

The volunteers from Jakarta capital city (total population of approximately 10 million) were on average 34 years (SD ± 10.5). The volunteers from Ende, Nangapanda and, East Nusa Tenggara villages (population size of roughly 87,269 representing the rural participants) were on average 34 years (SD ± 4.2). These villages are located roughly between 1,500 and 4,360 km from the urban site in Jakarta. The mean age of the participants was 34 years (SD ± 7.8) across all samples (Supplementary Table 1). The prevalence of good OHIS was found to be higher in urban women than in rural women (Supplementary Table 1).

**Figure 1:**
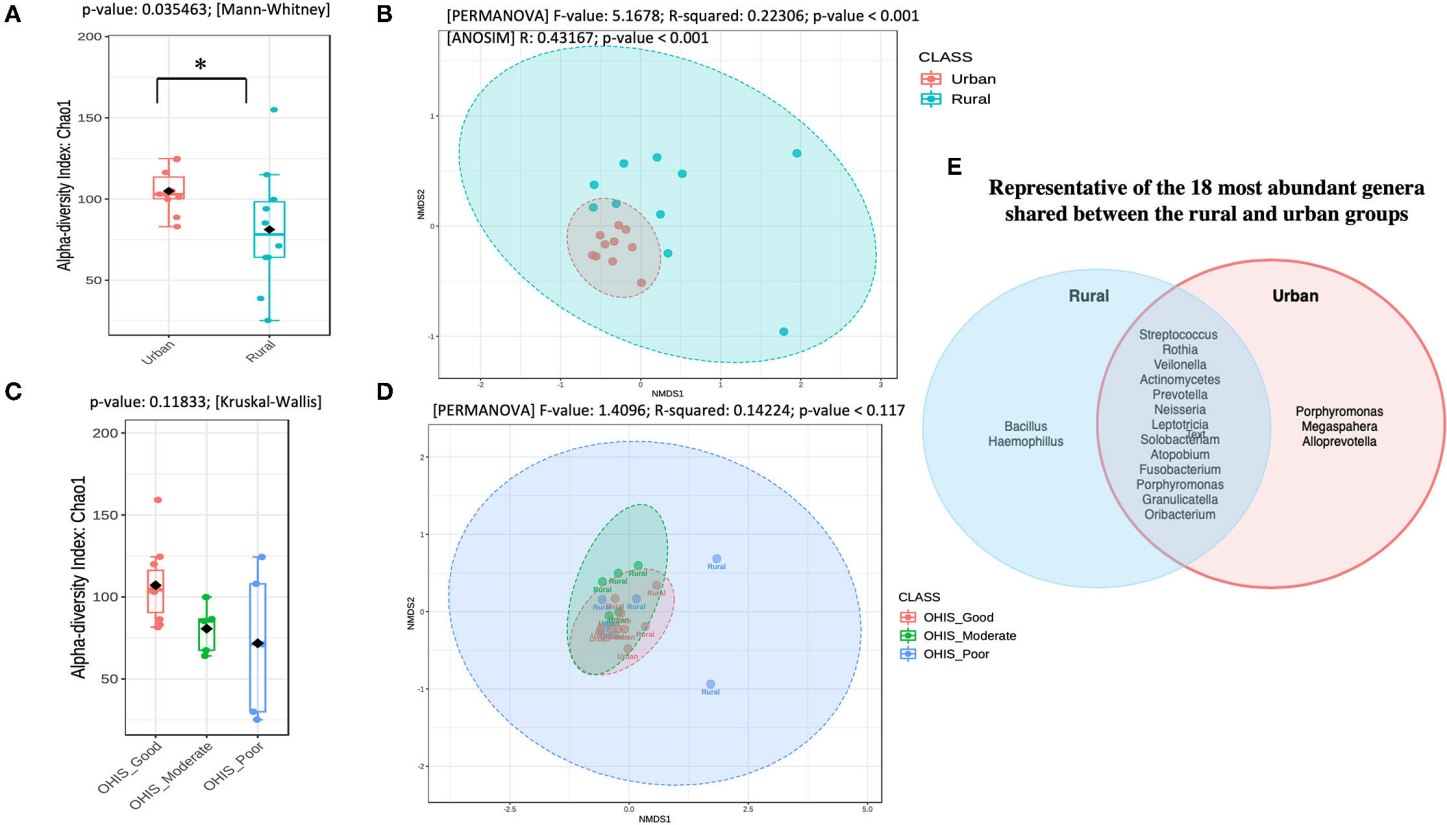
Overview of the oral microbiota diversity estimates and compositional structure between the two geographical studied populations **(A)** and **(C)** Alpha diversity analysis of the samples between the urban and rural subjects and stratified by oral hygiene indicator (OHIS); marginal boxplots describe the distribution of those values for the different groups; respectively. Statistical assessment was carried out using the two-tailed Mann–Whitney test and the Kruskal-Wallis test. Alpha-diversity indices. Richness index (Observed OTUs) and richness index (Chao-1) between women in the urban and rural oral microbiome. Letters indicate statistical differences between samples (*p* < 0.05). **(B)** and the **(D)** Microbial community structure of the two groups and the different level of OHIS oral hygiene indicators, respectively measured using the non-metric multidimensional scaling (NMDS) plot based on Bray–Curtis dissimilarity is drawn to display changes in microbial communities between two geographical locations. Inserted boxplots show the inter-site distance for samples between two locations and stratified by three different OHIS clinical indicators, respectively. Corresponding R2 and *p*-values from permutational multivariate ANOVA (PERMANOVA) tests were also shown. Letters indicate statistical differences between the samples ^*^p < 0.05 and ^***^p < 0.001. **(E)** Venn Diagram representing the 18 most abundant genera shared between the rural and urban groups.

To determine the oral bacterial dysbiosis associated between urban and rural populations, we assessed the microbial diversity and richness. Chao1-estimated the microbial richness to be significantly higher (Mann Whitney *U*-test, *p* = 0.035) in the oral samples of urban volunteers compared with the rural volunteers (Figure 1A and Supplementary Table 2). When accounted for the OHIS indicator of the volunteers from two geographic locations, there were no significant differences in species richness, although the bacterial species richness is considerably high in healthy (Good, Moderate) compared with poor oral hygiene Kruskal-Wallis chi-squared = 4.268, *p* = 0.118) (Figure 1C and Supplementary Table 2).

### Distinct Oral Microbiome Dysbiosis Between Urban and Rural Locations

The differences in the bacterial community structure between the rural and urban localities were visualized in an NMDS plot (Figure 1B, Supplementary Table 2). The urban and rural samples formed distinct clusters [PERMANOVA (*R*^2^ = 0.223; *p* < 0.001, [NMDS] Stress = 0.084). To determine if there were statistically significant differences in the bacterial community composition of the different geographic samples, an ANOSIM analysis was performed. A significant difference was observed in the bacterial community composition between the urban and rural localities (ANOSIM result: *R* = 0.43, *p* < 0.001). Further, a pairwise analysis using PERMANOVA showed that there was no significant difference among the oral OHIS hygiene status within each location (*R*^2^ 0.141; *p* < 0.119, [NMDS] Stress = 0.097) (Figure 1D). Meanwhile, the representative of the 18 most abundant genera shared between the rural and urban groups has been shown in Figure 1E.

### Taxonomic Alterations of the Oral Microbiome in Urban and Rural Localities

**Figure 2:**
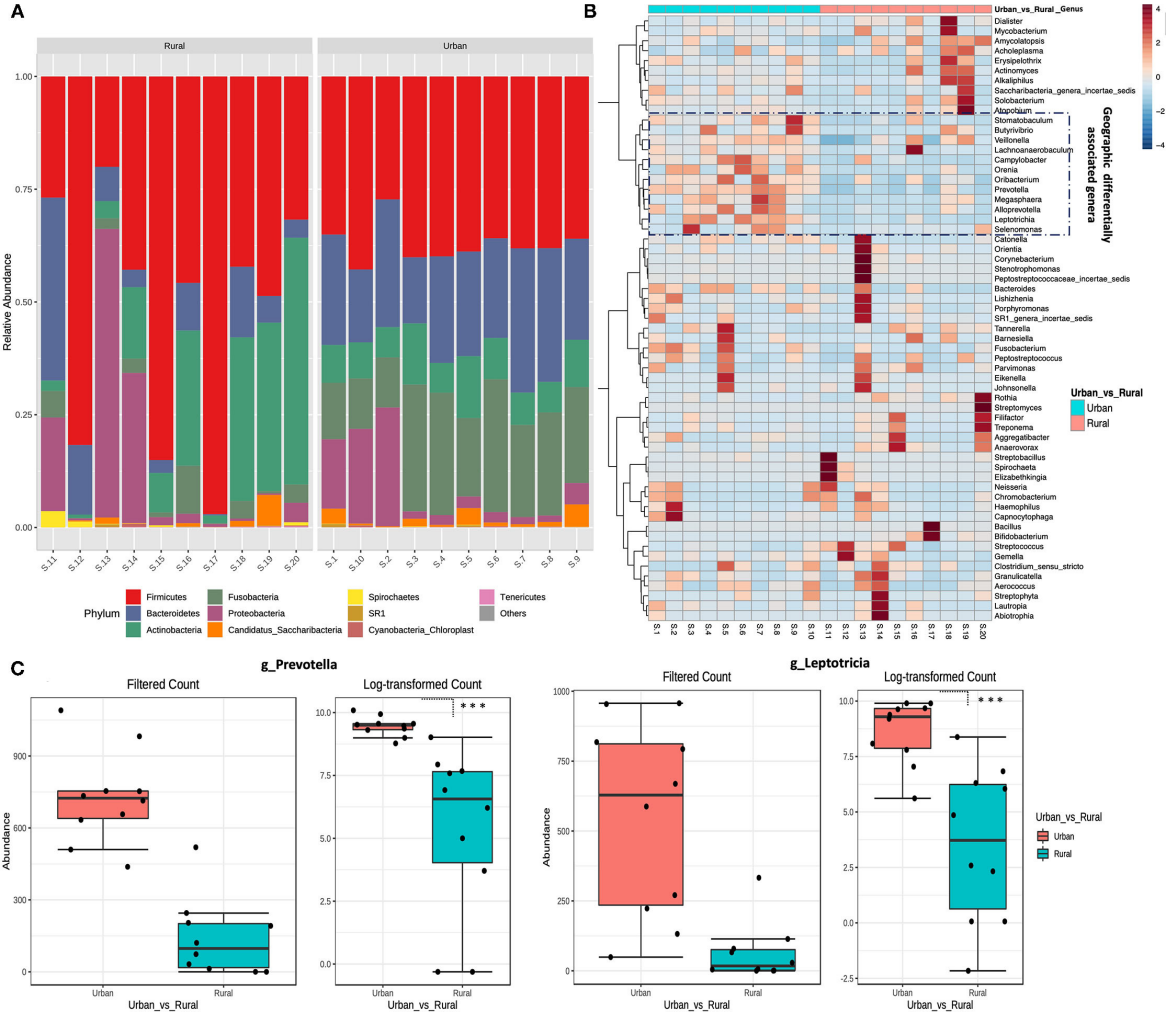
Taxonomical variation in oral microbiota between urban and rural women. **(A)** Relative abundance of the top 10 phyla between urban and rural subjects. **(B)** Heat map of the most abundant genera in each sample. The color intensity in each box indicates the relative percentage of a genus in each sample. The relative abundance data were z-scored normalized by row. To show the distribution of abundant operational taxonomic units (OTUs), the relative abundances of OTU data were normalized to have a mean of 0 and an SD of 1 (z-score normalization). The plot was made using the heatmap R package with the default parameters. The distribution of the OTUs is represented as the color intensity of each grid (blue, low abundance; red, high abundance). **(C)** Most significantly changed genera (genus *Prevetolla* and *Leptorticia*) relative to the abundance among the top 10 predominant genera (Genera with average relative abundance >3%) of the studied subjects shown. DefSeq2 analysis was used with the log2foldchange value, and multiple comparisons were corrected using the Benjamini–Hochberg correction (Q parameter = 0.1, FDR < 10%), ^***^adjusted *p* < 1.0 × 10^−4^.

The bacterial phylum and genera differing between every two groups were identified using DESeq2 analysis with adjustment for age (*p*.adj < 0.05). Overall, five distinct fungal phyla were detected in all the samples, based on sequences with relative abundances above 2% (Figure 2A). The majority of sequences were assigned to members of the phyla Firmicutes, which constituted 38% in the urban samples and 52% in the rural samples of the total relative abundance. The relative abundance of Bacteroidetes (23%) and Fusobacteria (20%) are higher in the urban compared with the rural location (11 and 3%, respectively), whereas Actinobacteria (19%) and Proteobacteria (13%) are comparatively higher in the rural locations (urban: 9 and 8%, respectively). Significant differences were detected at the phyla of Bacteroidetes and Fusobacteria (DefSeq [[I]]P[[/I]] <0.001; log2 fold-change values ranged from −3.4 to −2.7) enriched in urban oral samples, whereas the phyla of Firmicutes, Actinobacteria, and Spirochaetes (DefSeq *p* < 0.001; log2 foldchange values ranged from 3.5 to 1.4) were significantly higher in the rural oral samples.

The top 10 abundant genera in each sample were selected (a total of 35 genera for all the 10 samples), and their abundances were compared with those in other samples using heat map analysis (Figure 2B). In total, eight distinct bacterial genera (genera with average relative abundance >3%) were identified in both localities with *Streptococcus* constituting the majority of sequences (30%), followed by *Bacillus* (10%), *Rothia* (9%), *Veillonella* (8%), similar proportions of *Actinomycetes* and *Actinobacteria* (7%), *Neisseria*, and *Prevotella* (4%) in the rural location. Whereas *Prevotella* (21%) being the highest in urban location followed by *Leptotricia* (16%), *Streptococcus* (15%), *Veillonella* (12%), *Neisseria* (6%), *Actinomycetes* (5%), *Fusobacterium* (4%), and *Rothia* (3%). Significant differences were detected in the genera of *Prevotella* and *Leptotricia* in the urban population (DefSeq *P* <0.001; log2 fold-change values ranged from −3.4 to −2.7) (Figure 2C). We could not find a significant difference in the genera/Phylum change based on the OHIS of the volunteers from two localities.

### Ecological Drivers of Oral Microbiome Between Urban and Rural Localities

Besides the differences in the microbial composition mentioned above, we wonder if microbial interactions were also altered by urbanization, and thus constructed co-occurrence networks by SparCC between urban and rural populations. The SparCC method was used to generate these networks, as this has been shown to enable researchers to detect the linear relationships in a compositional dataset to a high degree of precision [39, 46] and explore the correlations that arise between OTUs that are indirectly connected in an ecological network.

**Figure 3:**
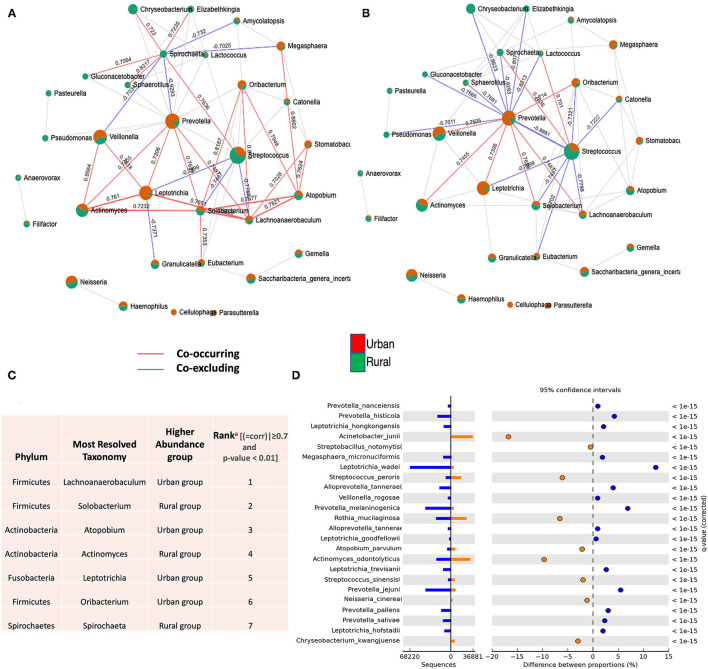
Co-occurrence network of oral microflora between two populations. SparCC correlations with *r* > 0.6 (or < -0.6–0) and *p* < 0.05 were shown. Each node represents a genus; the size of the node is proportional to the median relative abundance of the corresponding genus; connecting edges indicate correlations between them; the green and red colors represent the urban and rural population in the shared genus, respectively. The solid lines represent positive (Co-occurrence) (red) and negative (Co-excluding) (blue) correlations, *n* = 165. **(A)** SparCC correlation network between urban and rural oral microbiota with *r* > 0.6 (or < −0.6–0.) and *p* < 0.05. **(B)** SparCC correlation network in identified dominant oral core microbiome (Streptococcus and Prevetolla) between urban and rural localities with *r* > 0.7 (or <-0.7–0) and *p* < 0.05. **(C)** Identification and highest ranking of OTU contributions (^a^) to geographic co-occurrence pattern of oral microflora between two localities (>0.7 and *p* < 0.01). (See Supplementary Tables 3, 4). **(D)** Statistical differential bacterial taxonomical features regarding two geographical locations at the species level; illustrating most significant bacterial species in variations of different proportions among dominant core oral microbiome between two localities using STAMP tool. *P* < 0.01 to detect species with the most statistically significant differences filtered using a *q*-value of 0.05 and effective size of 0.05 threshold in STAMP.

For the entire bacterial network, a total of 165 pairs of significant and robust correlations were identified from 70 bacterial genera. Specifically, 165 pairs of positive correlations were identified from 65 bacterial genera, while 95 pairs of negative correlations were identified from 55 bacterial genera. After correcting for spurious correlations and FDR adjustments and filtering out the OTUs with >10 counts in the total samples of each group, significant and robust positive intra- and inter-correlations with the variations of bacterial community composition were identified in two localities (Figure 3A). Out of which, significantly higher correlations for seven OTU were observed, suggesting that the correlations between the same microbial pairs were similar between these two populations. (Positive |coefficient correlation (=corr)| ≥0.7 and *p* < 0.01 and negative correlations has (corr)|>-0.7 (*p* ≤ 0.01) (Figure 3C and Supplementary Table 3). These OTUs tend to represent copious numbers of the oral core microbiome in both localities (≤16% of relative abundance in urban and <8% of relative abundance in rural), exhibited higher abundance in the oral samples of the urban group classified as *Lachnoanaerobaculum, Atopobium, Leptotrichia*, and *Oribacterium*, whereas higher genera of *Solobacterium, Actinomyces*, and *Spirochaeta* were observed in the rural samples. As the network coverage in the co-occurrence pattern of these genera has higher positive correlations in relation to abundance [compared to negative correlations(co-excluding)] (Figures 3C,D), we could hypothesize that the oral bacterial assembly that belongs to these genera have a similar shared preference for their ecological interaction and psychological requirements in two localities. On the other hand, higher frequency of negative associations were observed among the dominant core oral microbiome [Streptococcus (rural) – (=corr)| ± >0.7 *p* < 0.05 and *Prevetolla* (urban) and ± >0.7 *p* < 0.05] (Figure 3B and Supplementary Table 4). Therefore, we could speculate that most of the bacterial assembly that represents the predominant genera (>20%) of the oral core microbiome in both localities have a unique tendency of species to co-occur the interconnections between its members to sustain the ecological balance.

### Differentially Abundant Biomarker Taxa Between Urban and Rural Localities

**Figure 4:**
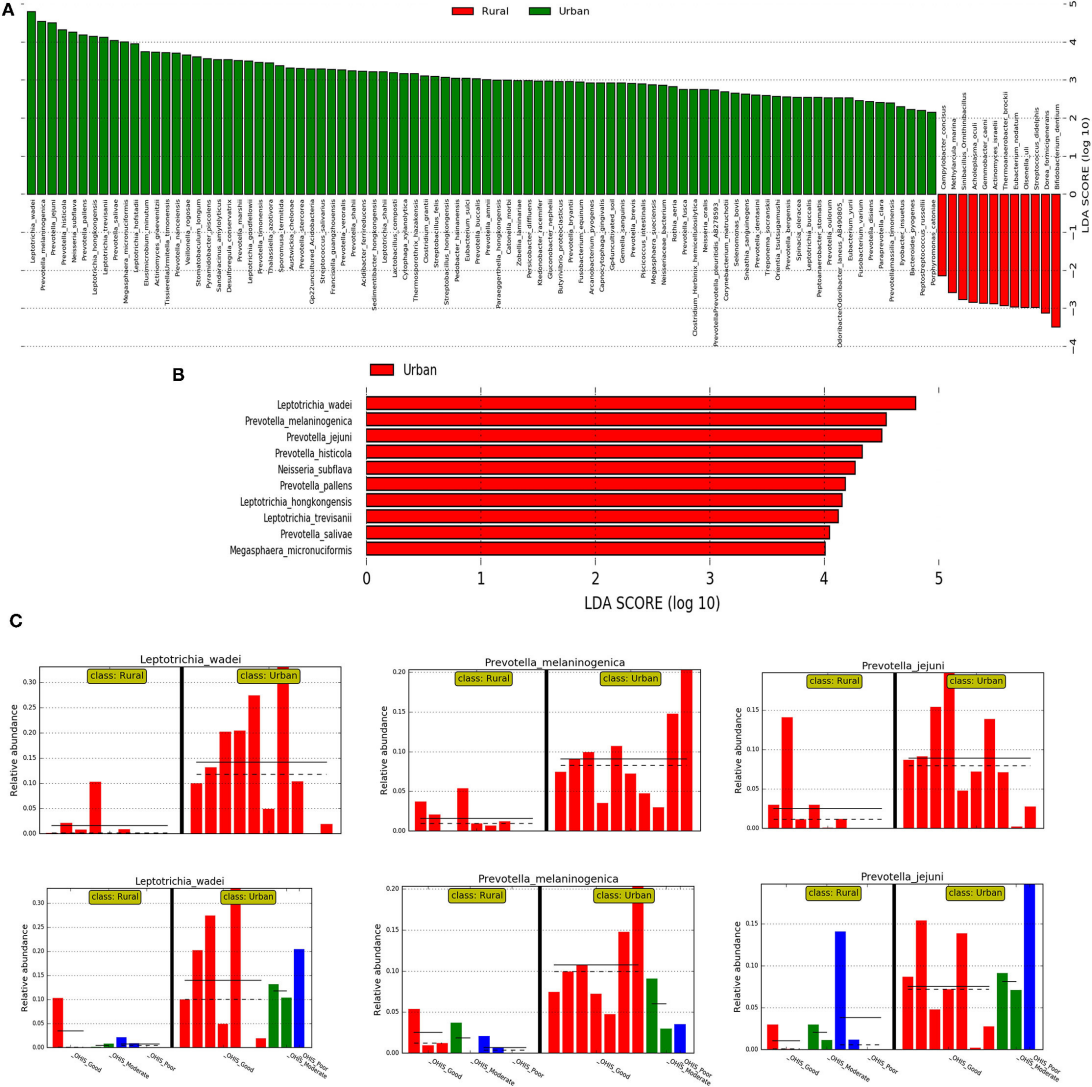
The results of the linear discriminant analysis (LDA) effect size (LefSe) analysis of the rural and urban oral microflora. Linear discriminant analysis effect size identifies taxa that are statistically different in biological states but also provides a magnitude of the effect. The histogram of the LDA scores was computed for the differentially abundant taxa between the rural and urban oral microflora. The bar size represents the effect of the size of specific taxa in the particular group at the species level. The LEfSe scores (LDA score) can be interpreted as the degree of consistent difference in relative abundance between features in the two classes of analyzed microbial communities. The histogram thus identifies which clades among all those detected as statistically and biologically differential explain the greatest differences between communities. **(A)** An LDA score value of 2.0 was chosen as a threshold for discriminative features **(B)** An LDA score value of 3.0 was chosen as a threshold for discriminative features. **(C)** Histogram of most significantly differentially abundant species of (in the 01 interval) *Leptotrichia wadei* and *Prevotella melamninogenica* in urban and rural samples and subclasses stratified by different levels of OHIS oral hygiene indicators represented by red (good), blues(moderate) and green (poor).

Then, we investigated the differentially abundant oral species associated with both urban and rural localities; and their differential abundance in relation to OHIS. Linear discriminant analysis and the LEfSe test for biomarkers were used to detect taxa that showed the strongest effect on group differentiation. The OTU level analysis uncovered a total number of 100 species (LDA score >2) (Figure 4A), 10 significantly urban-associated species (LDA score >4) from 3 genera, whereas, 12 rural-associated species (LDA score >3) from 5 genera, were detected. Out of which, only 10 clades can be considered as statistically and biologically differential, explaining the greatest differences between communities, which are exclusively identified in the urban population (Figure 4B). The most abundant urban-associated biomarker genera were *Prevotella, Leptotrichia*, and *Neisseria* at species level; *Leptotrichia wadei, Prevotella melamninogenica, Prevotella jejuni, Prevotella histicola, Neisseria_subflava, Prevotella pallens, Leptotrichia hongkongensis, Leptotrichia trevisanii, Prevotella salivae*, and *Megasphaera_micronuciformis* were driving the most significant differences. (Figures 4B,C and Supplementary Table 5). Irrespective of oral hygiene conditions, these taxonomical biomarkers were enriched in the urban female volunteers compared with the rural volunteers. The rural-associated biomarkers were dominated by the phyla Actinobacteria and Clostridia, Bifidobacterium, Streptococcus, and Actinomyces. The most abundant (LDA Score >2.8) species *Bifidobacterium dentium* and *Dorea formicigenerans, Actinomyces israelii*, and *Eubacterium nodatum* were exclusive to rural volunteers.

We then used the DESeq2 test based on variance transformed data with the negative binomial model (DESeq function with default arguments). This provided a ranking of the most differentially abundant taxa. With increased statistical rigor, the DESeq2 further supported in identifying the similar bacteria with the most significant changes in differential abundance between the urban and rural groups, belonging to genera *Leptotrichia* (*Leptotrichia wadei, Leptotrichia hongkongensis*, and *Leptotrichia_trevisanii*) and *Prevotella* (*Prevotella melaninogenica, Prevotella histicola, Prevotella pallens*, and *Prevotella salivae*).

## Discussion

Numerous 16S-based microbiome studies on oral microbiota in the literature have only conducted an analysis of the differential abundance of the taxa associated with various oral conditions [47, 48], in which geography was not considered as a potential factor structuring the oral microbiome. Our pilot study provides the first results showing the importance of geography on the oral microbiome dysbiosis in Indonesian populations. The results from this study suggest that the oral microbiome between these two studied regional populations could be structured by geography and lifestyle.

From the findings derived from these studies, the oral samples from the urban and rural groups had significantly different levels of richness and diversity. The oral microbiota of the urban localities was significantly richer in bacteria than rural localities. Further, the improving OHIS revealed a trend of increased bacterial alpha diversity, but these results were not statistically significant for any of the related parameters used in between-grade comparisons.

These results were further corroborated by the NMDS and PERMANOVA analyses, which not only show that both populations cluster distinctly and share just a few taxa, but that they have diverse bacterial community compositions consistent with rural and urban localities. No significant clear clustering was observed based on the oral hygiene status of the participants from the two regions. The clustering of a smaller proportion of the bacterial OTUs was categorized into discrete rural and urban groups within the Venn diagram. A larger percentage of OTUs were shared between the two populations, which may suggest that factors such as the environment, age, and diet may play a role in shaping the differences in a few OTU clusterings within this shared cohort.

In terms of our differential abundance findings, *Bifidobacterium dentium* was the most abundant rural-associated biomarker species found exclusively in the oral microbiomes of rural women. Oral *Bifidobacterium* has been recognized as a novel caries-associated bacterium. The predominant *Bifidobacterium* species in the oral cavity is *B. dentium*; known as an acid-tolerant opportunistic pathogen associated with dental caries and periodontal disease [49]. *Bifidobacterium dentium* is known to metabolize a much larger variety of carbohydrates than other *Bifidobacterium* species sequenced so far and greater than the range of oral streptococcal including *S. mutans*, and the ability to proliferate within active carious lesions [50]. Among the other reported periodontal disease bacteria, *Actinomyces israelii* and *Eubacterium nodatum* are significantly and exclusively identified in rural women. *Eubacterium nodatum* is a species of *Eubacterium* that has commonly been found in patients with chronic periodontal disease [51]. All the *Actinomyces* species etiologically involved in actinomycotic lesions in humans belong to the indigenous facultatively pathogenic microbiota of the human mucosal surfaces, especially in the oral cavity; *A. israelii* by far one of the most important agents of this disease [52, 53]. However, these reported bacteria were found only sporadically in the oral microbiome of the rural women studied here. In concert with these earlier studies; supporting the assumption that the mouth is not a reservoir for these putative caries and periodontal pathogens. Likewise, most of the differentially abundant biomarkers found in the urban group are mostly famous for their role as an opportunistic pathogen that is associated with oral diseases, halitosis, and poorer oral health consist of *Leptotrichia wadei, Prevotella melaninogenica, P. histicola, P. pallens*, and *Megasphaera micronuciformis* [54] which can be solely considered as urban taxonomic biomarkers (LDA score >4, DefSeq *P* < 0.001; log2 fold-change values ranged from −3.9 to −3.3) in our study. Previous studies have found the *P. pallens* group [55] and *L. wadei* as potential halitosis-associated bacteria, later being observed to have relatively higher abundance in halitosis tongue coating samples compared with healthy controls [56]. On the other hand, individuals with a high abundance of *P. histicola, P. melaninogenica*, several *Veillonella*, and *Streptococcus* species exhibited poorer oral health [54]. It was also shown that the prevalence of the genus *Megasphaera*, including *M. micronuciformis*, was elevated in cases of periodontitis [57]. Interestingly, Yang et al., studying the salivary microbiomes of caries-active and healthy individuals, found an association of *Prevotella* sp. (*P. histicola* among others) with caries-active individuals which was confirmed by Teng et al., who reported an increased abundance of various *Prevotella* species in the saliva of children with early childhood caries [58, 59]. Combined, the studies suggest that *Prevotella* can promote periodontitis by driving neutrophil recruitment *via* Th17 immune responses. In concert with this statement, previous studies have provided critical insights into inflammatory systemic diseases; like patients with rheumatoid arthritis (RA) without periodontitis have enrichment in periodontitis-associated bacteria, such as *Prevotella* (e.g., *P. melaninogenica, P. denticola, P. histicola, P. nigrescens, P. oulorum*, and *P. maculosa*) [60] and the increased levels of inflammatory mediators in the periodontal tissues of subjects with RA and other diseases are likely to be instrumental in determining periodontal disease activity later [61]. Therefore, changes in the systemic and local inflammation may disrupt microbial homeostasis and consequently increase bacterial pathogenicity and periodontal disease susceptibility [60]. It is evident that significantly higher proportions of *Prevotella* have an impact on the difference in oral microbiome perturbation in the studied urban women without periodontitis. Therefore, the presence of these organisms in the oral cavity coupled with underlying systemic diseases which are not investigated in the studied women could predispose individuals to oral diseases.

Interestingly, these poor oral health-related bacteria did not significantly drive the association with individual oral hygiene status in our finding. Therefore, the foregoing observations provided evidence that the dysbiosis of the oral microbiome in both localities is unaccounted by the oral hygiene status of the individuals, even though good oral hygiene status was found to be higher in the urban women than in the rural women based on the result of the demographic data of the study subjects. This inequality may be due to differences in oral health service distribution, utilization, and outcomes between urban and rural areas. For instance, people living in rural areas may lack education in the maintenance of oral care, compared with those living in urban areas [62]. They may also not have sufficient facilitates and access to oral healthcare [62–64]. Additionally, another factor that may contribute to the microbiome composition of the rural population is their habit of chewing betel leaf. As Iptika [65] found, there is a traditional rural belief that chewing betel leaf promotes strength of teeth, and provides the same level of pleasure as smoking, freshens the mouth, and relieves stress [64, 65]. However, there exists no scientific evidence concerning the benefits of chewing betel leaf. In fact, the betel chewing habit has been found to increase the risk of periodontal diseases, pre-malignant oral lesions, excessive tooth abrasion, and tooth fracture [62, 66]. Yet, people in rural areas continue the betel leaf chewing habit due to their social environment and belief, in addition to a lack of education and counseling from dental healthcare practitioners. Future studies should assess the correlation and significance among these genera, the betel leaf chewing habit, and the oral halitosis level [67]. Thereby, we could hypothesize that observed manifest shaped by different cultural factors, socio-economic status, dietary habits which accounts for the altered oral microbiome profiles in two geographical regions.

Further, to support this argument, microbial community-wide ecological interaction should be taken into account when considering the taxa abundance in oral samples from two different geographical locations. The positive and negative correlations detected in a co-occurrence network can describe the tendency of different species to co-occur specific to their niche. One of the most useful features of a co-occurrence network analysis is that hubs or keystone OTUs, which are highly associated taxa in a microbiome, can be identified [68]. In our finding, we observed the increasing strength of co-occurring patterns (higher number of positive correlations) in keystone OTUs of bacterial assembly among copious members of the core oral microbiome (≤16%) with few associated differential abundance results (genus *Leptotrichia*) between the rural and urban localities. This could confirm that identified keystone OTUs could have an impact on oral microbiome functioning, irrespective of the abundance parameters [69] with a similar shared preference for environmental conditions in both the rural and urban localities while the dominant oral core microbiome representing both localities have unique ecological interaction specific to its niche (comparatively higher number of negative correlations). Further, it is worthy to note that any comparison of the co-occurrence results in the different studies in the literature should be undertaken with caution: although current literature based on oral microbiome change based on geographic location is limited as the findings obtained may well be affected by methodological differences such as the different correlation values employed as cut-off points [70] or the use of different keystone OTU definitions [69].

In our study, the oral microbiome derived from tongue samples in both rural and urban groups was dominated by eight genera including three upper-level taxa, especially *Streptococcus* and *Prevotella*; respectively. It has been reported that the salivary microbiome appears to consist disproportionally of microorganisms from the tongue biofilm. Marger et al. reported that the papillate surface of the tongue harbor a microbiome skewed toward anaerobic bacteria such as *Prevotella* and *Veillonella*, whereas the ventral surface bears a microbiome rich in Streptococci and Gemella [71]. Consistent with the salivary microbiome, the contribution of tongue biofilm populations has been reported to contain several genera with the most prevalent or autochthonous being *Prevotella* and *Streptococcus* [2]. In concordance with these reports, three of these genera (*Prevotella, Streptococcus*, and *Veillonella*) including upper-taxa were found in both localities at the top of four higher proportions [2].

It is important to recognize that the present study had several limitations. First, only otherwise healthy individuals were enrolled in the study. Second, we used convenient sampling for this pilot study, hence the sample size of this cohort is not adequate to make firm conclusions. Third, future studies should examine a larger cohort that includes both genders, dietary habits, and other socioeconomic statuses.

## Conclusion

In conclusion, within the limitations of this study, our pilot study provides the first insight into the importance of geography on the oral microbiome in rural and urban locations in Indonesia. We found that the oral microbiome in women displays distinct patterns consistent with geographic locality. Our data could support the view that geographical specific changes of co-occurrence patterns in the dominant core oral microbiome assembly were unique to individual populations between urban and rural areas. The results of the biomarker analysis revealed several poor oral health-related bacterial taxa in both localities. In addition, some species might have a potential role in deteriorating oral hygiene which is different from the women in urban to rural areas, irrespective of the oral hygiene status of the individuals. Unique taxa being biomarker species in urban localities have potential consequences for this finding. This could be influenced by geographical specific dietary habits, cultural habits, and socioeconomic status. Future studies with a larger sample size are required to confirm the present findings.

## Data Availability Statement

The datasets presented in this study can be found in online repositories. The names of the repository/repositories and accession number(s) can be found below: NCBI SRA; PRJNA745286.

## Ethics Statement

The studies involving human participants were reviewed and approved by the Ethical Committee of the Faculty of Medicine, Universitas Indonesia (1060/UN2.F1/ETIK/PPM.00.02/2019). The patients/participants provided their written informed consent to participate in this study.

## Author Contributions

AW, CT, EB, and BB: contributed to conception and design of the study. AW and CT: performed the laboratory experiment and wrote the first draft of the manuscript. AD: organized the database. NU: performed the bioinformatic analysis and developing the manuscript. TA: performed the statistical analysis. All authors contributed to manuscript revision, read, and approved the submitted version.

## Funding

This study was supported in part by grants-in-aid from the Universitas Indonesia (PUTI Q1) with Grant No. BA-375/UN2.RST/PPM.00.03.01/2021, Jakarta, Indonesia and Trisakti University, Jakarta, Indonesia.

## Conflict of Interest

The authors declare that the research was conducted in the absence of any commercial or financial relationships that could be construed as a potential conflict of interest.

## Publisher's Note

All claims expressed in this article are solely those of the authors and do not necessarily represent those of their affiliated organizations, or those of the publisher, the editors and the reviewers. Any product that may be evaluated in this article, or claim that may be made by its manufacturer, is not guaranteed or endorsed by the publisher.
