# Supplementary material for: Diversity of Oral Microbiome of Women From Urban and Rural Areas of Indonesia: A Pilot Study
Source: Front Oral Health. 2021 Nov 29;2:738306. doi: 10.3389/froh.2021.738306 (PMC8757682; doi:10.3389/froh.2021.738306)
Supplement: Supplementary file 1 [file Data_Sheet_1.PDF]

Characteristic	Urban	Rural
Age(mean ± SD)	34.2 ± 10.5	34.7±4.2
Oral Hygiene Status [Green – Vermillion - Hirschman index-OHIS)(n)		
Good	7	3
Moderate	2	3
Poor	1	4

Supplementary Table 1 : Demographic and oral hygiene status evaluation (OHIS) of participants between urban and rural- The mean age of the participants was 34 years (SD ± 7.8) across all samples Oral Health index (OHI) for determining the soft sediments classified with microbial signatures denoted as no plaque (0), mild [1- (1/3) covered with soft plaque], moderate [2-(1/3-2/3) covered with soft plaque], bad [3- (>2/3) covered with soft plaque] according to Green – Vermillion - Hirschman index.

	Alpha Diversity						Beta Diversity	NMDS		ANONISM	
Sample name	Effective Sequences	Observed_Species	chao1	OHIS_status	chao1	Goods_Coverage	Sample name	MDS1	MDS2	MDS1	MDS2
Urban	40879	104	116.428571	OHIS_Good	120	99.7546765	Urban	-0.1052	-0.19167	-0.1052	-0.19167
Urban	33057	79	103	OHIS_Good	104	99.9080037	Urban	0.0089381	-0.51479	0.0089381	-0.51479
Urban	29776	97	99.875	OHIS_Moderate	99.875	99.7853419	Urban	-0.27465	0.0076056	-0.27465	0.0076056
Urban	34008	77	105	OHIS_Good	105	99.7546765	Urban	-0.60361	-0.264	-0.60361	-0.264
Urban	76297	92	124.909091	OHIS_Good	124.5	99.9080037	Urban	-0.18456	-0.030289	-0.18456	-0.030289
Urban	64547	85	83	OHIS_Good	83	99.8773382	Urban	-0.55861	-0.27477	-0.55861	-0.27477
Urban	47846	80	124.333333	OHIS_Poor	124.333333	99.9080037	Urban	-0.50712	-0.083071	-0.50712	-0.083071
Urban	55699	88	88.75	OHIS_Good	86.3333333	99.6933456	Urban	-0.33231	-0.13972	-0.33231	-0.13972
Urban	48296	77	101.111111	OHIS_Moderate	86.5454546	99.6013493	Urban	-0.44555	-0.16521	-0.44555	-0.16521
Urban	39985	82	102.875	OHIS_Good	103.111111	99.8466728	Urban	-0.35101	-0.32055	-0.35101	-0.32055
Rural	8287	31	38.75	OHIS_Poor	30	99.8160074	Rural	1.7866	-0.95732	1.7866	-0.95732
Rural	5213	28	25.2	OHIS_Poor	25.2	99.9386691	Rural	1.9506	0.66077	1.9506	0.66077
Rural	39965	81	155	OHIS_Good	159.2	99.7546765	Rural	0.33997	-0.24698	0.33997	-0.24698
Rural	41318	99	94	OHIS_Poor	108	99.7853419	Rural	0.24613	0.10634	0.24613	0.10634
Rural	8058	92	99.75	OHIS_Good	81.5833333	99.724011	Rural	0.51892	0.47465	0.51892	0.47465
Rural	34537	93	115	OHIS_Good	104.428571	99.8466728	Rural	-0.30659	0.20253	-0.30659	0.20253
Rural	4707	89	85.2307692	OHIS_Moderate	85.2307692	99.8160074	Rural	-0.21142	0.56848	-0.21142	0.56848
Rural	36977	70	71.1764706	OHIS_Poor	71.1764706	99.8466728	Rural	-0.59258	0.16962	-0.59258	0.16962
Rural	39659	67	64.2	OHIS_Moderate	67.5	99.6933456	Rural	-0.5818	0.37463	-0.5818	0.37463
Rural	2448	73	64.0769231	OHIS_Moderate	64.0769231	99.724011	Rural	0.20384	0.62376	0.20384	0.62376

Supplementary Table 2: Alpha diversity indices between urban and rural populations in each group at 97% identity presented on the left side of the table. the non-metric multidimensional scaling (NMDS) plot based on Bray–Curtis dissimilarity on two axis in each group at the OTU level at 97% identity presented on right side of the table.

Spearman's rank correlation - Positive correlation-coefficient correlation (=corr)|>0.8 and p-value < 0.01				
Taxon_Genus	Taxon_Genus	(|coefficient correlation (=corr)	P.Value	FDR
Lachnoanaerobaculum	Solobacterium	0.9281	0.0099	0.0891
Atopobium	Lachnoanaerobaculum	0.8723	0.0099	0.081
Leptotrichia	Actinomyces	0.8634	0.0099	0.0693
Parasutterella	Sphaerotilus	0.8512	0.0099	0.225
Pasteurella	Pseudomonas	0.8509	0.0099	0.138
Actinomyces	Veillonella	0.8434	0.0099	0.075
Leptotrichia	Lachnoanaerobaculum	0.8379	0.0099	0.069
Atopobium	Solobacterium	0.8345	0.0099	0.089
Oribacterium	Solobacterium	0.8162	0.0099	0.089
Cellulophaga	Parasutterella	0.8132	0.0099	0.225
Spearman's rank correlation - Negative correlation-coefficient correlation (=corr)| -(0.7-0.79) and p-value ≤ 0.01				
Amycolatopsis	Chryseobacterium	-0.701	0.0099	0.176
Lachnoanaerobaculum	Elizabethkingia	-0.7105	0.0099	0.100
Lachnoanaerobaculum	Delftia	-0.7151	0.0198	0.105
Actinomyces	Elizabethkingia	-0.7258	0.0099	0.075
Solobacterium	Elizabethkingia	-0.728	0.0099	0.074
Stomatobaculum	Delftia	-0.7285	0.0099	0.148
Streptococcus	Oribacterium	-0.7299	0.0198	0.198
Spirochaeta	Leptotrichia	-0.7415	0.0099	0.069
Atopobium	Delftia	-0.7489	0.0099	0.148
Leptotrichia	Streptococcus	-0.7559	0.0198	0.198
Actinomyces	Spirochaeta	-0.7916	0.0099	0.075

Supplementary Table 3 : Co-occurrence network of keystone oral microflora between urban and rural populations. Only correlations with a R-corr absolute value greater than 0.3 and p-value less than 0.05 were plotted. We considered a valid co-occurrence event to be a robust correlation if the Spearman's correlation coefficient was both >0.6 (or <−0.6) and statistically significant (P < 0.05). Significant higher positive correlation was observed with r >0.7 (or <−0.7) and P < 0.01

**Spearman's rank correlation - Positive correlation-coefficient correlation (=corr)|0-6-0.7 and p-value < 0.05**			
**With Streptococcus**	**Correlation**	**P-Value**	**FDR**
Sphaerotilus	0.7862	0.0198	0.198
Spirochaeta	0.6895	0.0396	0.2376
Chryseobacterium	0.6873	0.0396	0.2376
Elizabethkingia	0.6858	0.0198	0.198
Delftia	0.6536	0.0495	0.27844
Granulicatella	0.6148	0.0396	0.2376
Cellulophaga	0.6031	0.0198	0.198
**Spearman's rank correlation - Negative correlation-coefficient correlation (=corr)| -(0.6-0.5) and p-value ≤ 0.05**			
Actinomyces	-0.6425	0.0198	0.198
Solobacterium	-0.685	0.0198	0.198
Prevotella	-0.692	0.0297	0.2376
Catonella	-0.6921	0.0198	0.198
Lachnoanaerobaculum	-0.6976	0.0198	0.198
Oribacterium	-0.7299	0.0198	0.198
Leptotrichia	-0.7559	0.0198	0.198

**Spearman's rank correlation - Positive correlation-coefficient correlation (=corr)|0-6-0.7 and p-value < 0.05**			
**With Prevetolla**	**Correlation**	**P-Value**	**FDR**
Leptotrichia	0.7794	0.0099	0.1782
Actinomyces	0.7513	0.0297	0.1782
Veillonella	0.6597	0.0198	0.1782
Lachnoanaerobaculum	0.6452	0.0198	0.1782
Solobacterium	0.6225	0.0297	0.1782
Atopobium	0.5995	0.0198	0.1782
Megasphaera	0.5719	0.0297	0.1782
**Spearman's rank correlation - Negative correlation-coefficient correlation (=corr)| -(0.7-0.6) and p-value ≤ 0.05**			
Granulicatella	-0.6163	0.0297	0.1782
Spirochaeta	-0.618	0.0099	0.1782
Elizabethkingia	-0.6358	0.0099	0.1782
Delftia	-0.6612	0.0099	0.1782
Streptococcus	-0.692	0.0297	0.1782

Supplementary Table 4 : Co-occurrence network of in keystone oral microflora between urban and rural populations. SparCC correlation network in identified dominant oral core microbiome (Streptococcus and Prevetolla) between urban and rural localities with r >0.7 (or <−0.7-0) and p < 0.05

Species	LEfSe			LEFSe With OHI status		DefSEq2			
	Log2LDA Score	P value	Condition	Log2LDA score	P value	log2FC	lfcSE	Pvalues	FDR
Leptotrichia_wadei	4.79882772	0.00695109	Urban	4.807514003	0.006951093	-3.398	1.0682	0.001467	0.028809
Prevotella_melaninogenica	4.54285532	0.00109084	Urban	4.551935858	0.001090835	-2.5443	0.7703	0.00095659	0.024393
Prevotella_jejuni	4.50616832	0.01133331	Urban	4.497681627	0.011333306	-	-	-	-
Prevotella_histicola	4.33167238	0.00044395	Urban	4.349283715	0.000443952	-3.1435	0.82978	0.00015168	0.0077357
Neisseria_subflava	4.27128428	0.03945726	Urban						
Prevotella_pallens	4.18730909	0.00079789	Urban	4.142584512	0.000797893	-3.5769	0.90599	7.88E-05	0.0060267
Leptotrichia_hongkongensis	4.15694383	0.00145086	Urban	4.154219956	0.001450862	-2.7995	0.84678	0.00094611	0.024393
Leptotrichia_trevisanii	4.1255422	0.01133331	Urban	-	-	-3.1736	1.0704	0.0030286	0.042125
Prevotella_salivae	4.04668327	0.00057227	Urban	4.035391404	0.000572273	-2.6307	0.90667	0.0037133	0.044315
Megasphaera_micronuciformis	-	-	Urban	4.011421956	0.008860634	-	-	-	-
Leptotrichia_goodfellowii	-	-		-	-	-3.9856	0.93045	1.84E-05	0.0028145
Bifidobacterium_dentium	3.49062965	0.00845578	Rural	3.4951064	0.008455784	-3.9856	0.93045	1.84E-05	0.0028145
Dorea_formicigenerans	3.12036838	0.02205578	Rural	-	-	-	-	-	-
Streptococcus_didelphis	2.97239952	0.04122683	Rural	-	-	-	-	-	-
Olsenella_uli	2.97161265	0.02283613	Rural	-	-	-	-	-	-
Eubacterium_nodatum	2.95570582	0.02946554	Rural	-	-	-	-	-	-
Thermoanaerobacter_brockii	2.92260279	0.02283613	Rural	3.227721949	0.022836126	-	-	-	-
Actinomyces_israelii	2.86780935	0.02205578	Rural	-	-	-	-	-	-
Gemmobacter_caeni	2.86690965	0.02411582	Rural	-	-	-	-	-	-
Acholeplasma_oculi	2.84069652	0.00145086	Rural	-	-	-	-	-	-
Sinibacillus_Ornithinibacillus	2.76278015	0.04536065	Rural	-	-	-	-	-	-
Methylarcula_marina	2.58189394	0.01789227	Rural	-	-	-	-	-	-
Campylobacter_concisus	2.14339738	0.0382974	Rural	-	-	-	-	-	-

Supplementary Table 5: Phylotypes Differentially Represented in oral microflora of women between urban and rural location calculated using the DESeq2 and Linear discriminant analysis (LDA) effect size (LefSe) analysis found overall 5 OTUs in studied cohort that were significantly differentially abundant and passed FDR (significance highlighted in yellow colour).
